# Do patient-reported outcome measures measure up? A qualitative study to examine perceptions and experiences with heart failure proms among diverse, low-income patients

**DOI:** 10.1186/s41687-022-00410-9

**Published:** 2022-01-15

**Authors:** Jonathan Davis, Kristan Olazo, Maribel Sierra, Michelle E. Tarver, Brittany Caldwell, Anindita Saha, Sarah Lisker, Courtney Lyles, Urmimala Sarkar

**Affiliations:** 1 Division of Cardiology, Department of Medicine, Zuckerberg San Francisco General Hospital, University of California, 1001 Potrero Avenue, 94110 San Francisco, CA USA; 2 Division of General Internal Medicine, Department of Medicine, Zuckerberg San Francisco General Hospital, University of California San Francisco, 1001 Potrero Avenue, Building 10, Ward 13, San Francisco, CA 94110 USA; 3 Center for Vulnerable Populations, Department of Medicine, University of California, San Francisco, CA USA; 4 Food and Drug Administration, Center for Devices and Radiological Health, Silver Spring, MD USA; 5 Department of Epidemiology and Biostatistics, University of California, San Francisco, CA USA

**Keywords:** Heart failure, Qualitative research, Outcomes research

## Abstract

**Background:**

The Kansas City Cardiomyopathy Questionnaire (KCCQ) is a Patient-Reported Outcome Measure (PROM) used to evaluate the health status of patients with heart failure (HF) but has predominantly been tested in settings serving predominately white, male, and economically well-resourced populations. We sought to examine the acceptability of the shorter version of the KCCQ (KCCQ-12) among racially and ethnically diverse patients receiving care in an urban, safety-net setting.

**Methods:**

We conducted cognitive interviews with a diverse population of patients with heart failure in a safety net system to assess their perceptions of the KCCQ-12. We conducted a thematic analysis of the qualitative data then mapped themes to the Capability, Opportunity, Motivation Model of Behavior framework.

**Results:**

We interviewed 18 patients with heart failure and found that patients broadly endorsed the concepts of the KCCQ-12 with minor suggestions to improve the instrument’s content and appearance. Although patients accepted the KCCQ-12, we found that the instrument did not adequately measure aspects of health care and quality of life that patients identified as being important components of managing their heart failure. Patient-important factors of heart failure management coalesced into three main themes: social support, health care environment, and mental health.

**Conclusions:**

Patients from this diverse, low-income, majority non-white population experience unique challenges and circumstances that impact their ability to manage disease. In this study, patients were receptive to the KCCQ-12 as a tool but perceived that it did not adequately capture key health components such as mental health and social relationships that deeply impact their ability to manage HF. Further study on the incorporation of social determinants of health into PROMs could make them more useful tools in evaluating and managing HF in diverse, underserved populations.

**Supplementary Information:**

The online version contains supplementary material available at 10.1186/s41687-022-00410-9.

## Background

Heart failure (HF) is a common, progressive, fatal disease with a prevalence expected to increase by 40% over the next 10–15 years [1]. In the United States, HF incidence, morbidity, and mortality are worse for African American, Hispanic, and other minority populations as compared to Caucasians [2, 3]. Patient-reported outcome measures (PROMs) are important tools to evaluate HF treatments and are associated with clinical HF outcomes [4]. Routine use of PROMs can significantly benefit the provider’s ability to provide personalized, tailored patient care [5]. The Kansas City Cardiomyopathy Questionnaire (KCCQ) is a 23-item questionnaire qualified as a medical device [6] and drug development tool by the FDA [7]. The KCCQ includes items about daily functioning, symptoms, and quality of life (QoL) in patients with HF. Clinical trials often employ the validated KCCQ as a clinical endpoint, due to its ability to predict hospitalization and death [8]. The shorter version of the KCCQ—the KCCQ-12—retains the psychometric properties of the full KCCQ, but is more feasible to implement as it minimizes the response burden patients experience [9]. The formative studies used to develop and validate the KCCQ and KCCQ-12 drew from predominantly White and male populations, similar to many clinical trials and observational studies in HF [8, 10]. We lack a complete understanding of how patients of different racial/ethnic backgrounds respond to items (questions) within the KCCQ [11]. In addition, providers, payers, and policymakers increasingly recognize social determinants of health (SDOH) as direct contributors to HF morbidity and mortality [11, 12]. Despite this, PROMs like the KCCQ and KCCQ-12 do not include information about SDOH and its impact on HF symptoms or self-management.

The goal of this project is to address this health equity gap through the examination of how a racially/ethnically diverse, low-income sample of patients with HF perceive the KCCQ-12. This qualitative inquiry explores how this PROM resonates with diverse patients, including how lived experiences directly impact HF self-management and influence their symptom reporting.

## Methods

### Study setting and patients

This study enrolled patients receiving outpatient primary or cardiology care at an academic, urban, safety-net system that serves one in eight San Franciscans regardless of immigration or insurance status. The San Francisco Health Network’s (SFHN) Cardiology Clinic at Zuckerberg San Francisco General Hospital (ZSFG) delivers over 4,000 annual visits. Patients have primarily Medicaid or Medicare or are uninsured. Patients were eligible if they met all of the following criteria: (1) spoke English; (2) were between the ages 24–85; (3) received care at primary care or Cardiology clinics at the ZSFG location; (4) diagnosed with HF; and (5) did not have a significant cognitive or visual impairment. We invited eligible individuals to participate in person at ZSFG or by mail, email, or phone. All study visits beginning April 2020 were virtual given the COVID-19 pandemic. Patients provided consent and self-reported demographics. Those who completed an interview with our study team received $25 in cash or gift card for their time. The UCSF Institutional Review Board (IRB) (#18-26769) and the FDA IRB (2019-CDRH-019) approved this study.

### Interviews

To characterize and understand how a diverse sample of HF patients interpreted items and responses within the KCCQ, we presented the KCCQ-12 [9], an abbreviated version of the KCCQ questionnaire. Compared to the KCCQ, the KCCQ-12 omits the stability, symptom burden, and symptom efficacy domains plus items from the physical limitation, quality of life, and social limitations domains. The KCCQ-12 remains a valid and reliable tool to measure a patient’s HF-related health status and how it impacts QoL, physical, and social activities [9]. The KCCQ-12 was thus used to be better integrated into clinic workflow. All 18 patients received the KCCQ-12 on paper, and of those patients, six (33%) also received the KCCQ-12 tablet-adapted version. For exploratory purposes, six (33%) also received an adapted version with a lower reading level on paper (see Table 4 for Flesh Kincaid (FK) score). The KCCQ-12 and reading level-adapted KCCQ-12 were printed on 8.5 × 11-inch standard white paper and used 10-pt Arial black font. The study team asked patients for feedback on both content and layout of the KCCQ-12 (e.g., how the KCCQ-12 was presented, readability and font size).

Trained interviewers (KO, MS, SL) conducted cognitive interviews using a semi-structured interview guide (Additional file 1). Cognitive interviewing is a qualitative research method that is used to understand whether questionnaires and survey questions work as intended [13]. There are two approaches that can be used to conduct a cognitive interview—think-aloud and verbal probing. Think-aloud is a technique in which participants are instructed to say anything that comes to mind as they go through the survey. Verbal probing is another technique in which the interviewer follows up with another question to elicit a more detailed response from the participant [13, 14]. Our team used both the think-aloud and verbal probing methods concurrently [13]. The interviews took between 60 and 90 min. Study staff audio-recorded interviews with participant permission. The interviews were professionally transcribed for analysis.

### Analysis

According to best practices in qualitative research, we conducted data analysis in parallel with data collection, using the method of constant comparisons. We reached thematic saturation—the point at which no new themes emerged from the interviews—after analyzing 10 interviews and completed data analysis for the remaining two interviews. For the reading level-adapted KCCQ-12, we reached thematic saturation after four of the six interviews.

We utilized Dedoose qualitative analysis software (Los Angeles, CA) to code transcripts [15]. Using an inductive and deductive approach, we coded qualitative interviews to reduce code bias. Two study coders (KO, MS) collaboratively analyzed transcripts to lower variability between interviewers and to support consistent data cleaning.

For the deductive coding, we applied the behavioral Capability, Opportunity, Motivation model of Behavior (COM-B) model [16] to explore the open-ended participant comments about the behaviors of heart failure symptom management/self-management. Michie et al. describes the *capability* to entail both the patient’s psychological and physical capacity, plus having the necessary knowledge and skills to engage. *Opportunity* involves those external factors that impact behavior. Finally, *motivation* includes those factors that prompt and guide behavior, including conscious and unconscious processes. We developed a list of thematic codes based on the interview guide domains and topics then transcripts were re-reviewed along with notes and memos to determine broader thematic codes. Through this process, we developed sub-themes to capture participant experiences.

## Results

In total, we conducted 18 qualitative interviews with heart failure patients from April 2019 to September 2020. The mean age was 52 years, 33% were women, and 78% were non-White. Complete baseline demographics are in Table 1.

**Table 1:** Cognitive interview participant characteristics

Characteristics	Overall (N = 18)
Mean age (range)—years	52 (11)
Age categories—no. of patients^a^ (%)	
18–35 years	1 (6)
36–45 years	4 (24)
46–55 years	6 (35)
≥ 56 years	6 (35)
Sex—no. of patients (%)	
Female	6 (33)
Male	12 (67)
Ethnicity—no. of patients (%)	
Hispanic or Latino	3 (17)
Not Hispanic or Latino	15 (83)
Race—no. of patients^a^ (%)	
White or Caucasian	3 (22)
Black or African American	6 (33)
Asian	1 (6)
Native Hawaiian or Other Pacific Islander	2 (11)
Other or mixed	5 (28)
Highest education completed—no. of patients (%)	
High school diploma or GED	4 (22)
Some college	8 (44)
Associates or bachelor’s degree	3 (17)
Master’s, doctoral, or other professional degree	2 (11)
Other	1 (6)
Comfort with filling out medical forms—no. of patients (%)	
Not at all	0 (0)
A little bit	1 (6)
Somewhat	4 (22)
Quite a bit	5 (28)
Extremely	8 (44)

^a^Missing age and race for one participant

Study patients shared a range of experiences managing their heart failure condition as well as a variety of feedback on the KCCQ-12 [original (n = 12) and tablet (n = 6) and reading level-adapted (n = 6) versions]. Table 2 summarizes feedback on the questionnaire including suggested changes.

**Table 2:** KCCQ-12 feedback

Suggested change/explanation	N patients (N changes)	Examples of suggested changes
Question phrasing/Challenges with relevance or interpretation of specific questions	5 (9)	“Yes. I will reword it like how many times a day do you have to really make yourself comfortable by doing certain things to make yourself comfortable with pillows, standing, sitting, stuff like that.”—ptid129 “If you had to spend the rest of your life with a heart failure, what way is right? No, why would I want, I’ll read that over. If you had to spend the rest of your life with your heart failure, the way it is right now, they’re saying how would you go about it, how are you feeling right now? How would you feel about spending the rest of your life right there? I will go, ‘anything is better than dead.’ [Laughter] If you’re dead you ain’t feeling nothing, so I would go this mostly dissatisfied, mostly satisfied, completely dissatisfied, not at all satisfied. I don’t like this.”—ptid129 “The word “practical” is very important. What practical effects do showering, walking or hurrying, jogging have on your day-to-day?”—ptid400 “I would, first of all, change the words to—from “enjoy your life” to “quality of life.”—ptid400
Response phrasing/Patients desired alternative response options	5 (5)	“How many times did you feel too out of breath to do what you want? I’m just going with less than once a week, but actually, if it was less than 2 weeks, I would change it to less than 2 weeks, but I can’t use the word “never,” because “never” would imply that I’m not having that issue, you know, when it then becomes an issue, yeah.”—ptid88 *Response to Question 4* “It’s not really moderately. It could be a lot of different things. ‘Slightly.’”—ptid129 *Response to Question 1* “I would say three or more times per day.”—ptid190 *Response to Question 5*
Appearance changes/Patients desired aesthetic changes to formatting to improve the readability and engagement with the questionnaire	3 (3)	“Keep it Sans—Sans Serif and increase the font.”—ptid400 **“**Put it on some colorful paper. Change the paper.”—ptid129 “Maybe it could be a bigger lettering but other than that, it’s good.”—ptid345

Applying the COM-B framework [16] to the interviews, we identified several key issues for the self-management of heart failure (Table 3). In the capability domain, both physical issues related to medication adherence and psychological issues related to both general HF disease management and their experience with health care providers affected self-management. Patients were concerned about their ability to practice HF self-management behaviors such as taking medications, exercising, and maintaining a balanced diet. They reported unclear knowledge of key behaviors and actions related to HF self-management, which correspond with their ability to understand health information. In the motivation domain, themes related to beliefs about their own identity and role as a patient with HF, emotions, and attitudes toward heart disease, and their goals for HF management emerged. Items in the opportunity domain intersected with the motivation domain, namely their social support and physical environment such as living environment. Both strongly impacted their ability and desire to manage their HF. Environment—home, neighborhood, work, and even clinic—repeatedly emerged as a major SDOH that touched every aspect of their HF management.

**Table 3:** Mapping quality of life and HF management to the COM-B framework

COM-B components	Theme	Illustrative quote
*Capability*		
*Physical* (Skills, abilities acquired through practice)	Environment/competing demands for HF management	“I used to have one client in Beverly Hills who I would help clean their home once a week and I really like that they like me, I like them and I did a very nice, thorough obsessive–compulsive job of cleaning their place but I can’t do it any longer because there’s too much bending down.”—ptid51
*Psychological* (Knowledge, memory, attention, decision process, behavioral regulation)	Mental health barriers	“Nobody focus about what is a relation between my psychiatry and my illness, and I know my psychiatry caused the damage in me because I have a lot of sadness. A lot of psychiatry problem damaged my head, caused me—I feel the pain when I am sad more than when my diabetes is high or cholesterol is a high.”—ptid327 “If you’re not in tune with yourself mentally and you’re getting down upon yourself, there’s no way you’re going to stick to a medical regimen. Most of them (health care providers) do understand it but a lot of them haven’t tied the two in together yet, that there’s a physical aspect and there’s also a mental aspect to it.”—ptid350 “Anxiety is one thing also that has affected me. I mean, anxiety has a major impact on my life, but now since I have a heart failure, it’s even more.”—ptid395
*Opportunity*		
*Physical* (Environment context and resources)	Environment/competing demands for HF management	“I’m living in a place where it’s not secured. I’m seeing all this drug addiction around me and seeing all the stuff around me, and it—I stay in line, and it’s just—this is not like how I used to see it. This is getting really, really bad to where you go outside and all you see is feces on the ground. People don’t use the bathroom. People selling drugs. I mean, this is like an open market to do that you want to do now. Everybody who’s crazy is down there. It’s like for mental people with problems. Everybody down there got mental problems and they know where they’re putting them. They put them in this one area and anybody can come there. They come from all over the world. They come down to you, and you get down there and you can get stuff easy, and I’m trying my best because I was addict for about 30 years and I got like—going on 11 years now—clean and sober.”—ptid129 “Well, it’s San Francisco, you have three stories. Here is a one flat in Oakland but they built everything upwards instead of outwards, so yes, it limits me because walking up and down the flights of stairs. Whoever designed my home is an idiot because there’s no rest room on the first floor, so you have to go to the second and third to use the bathroom. Yes, doing household chores, we’re going to go with severely limited because the kitchen is also on the second. Whoever designed that was a complete buffoon, so I’m just going to leave it at that. You put the dining room and the kitchen on the second floor, you put a living room and a family room on the first and you put all the bedrooms upstairs. I didn’t design it but I’m just leaving it at that. [Laughter] It limits me because I have a hard time walking up and down the stairs. It limits me from being in certain parts of the house. ”—ptid350 “And ever since the fires, I haven’t been able to exercise but I did take up walking and—and, you know, very [Unintelligible] jogging like power walking, really fast walking but I haven’t been able to exercise since the fires.”—ptid264
*Social* (Influences such as social pressure, norms, conformity and social comparisons)	Social support	“There are friends that’ll hear me out and give me their opinion about things. I’m glad I have networks of people that I can basically talk to. You need to talk somebody about it.”—ptid375 “I can't do any house work, so I have someone to come to do that for me.”—ptid190 “My girlfriend, she helps me with like managing my salt and my food because I’m a salt person.”—ptid133 “I’m now able to do like one-tenth of what I was able to do before, so the other nine-tenths, I’ve had to create, delegate, reassign all the those things that I used to do, and then the same goes for me in that I now have someone that comes in to help me to do those—that nine-tenth that I can’t do, and then the visiting family and friends, that’s pretty much gone out the window coupled by the COVID quarantine.”—ptid400
*Motivation*		
*Automatic* (Emotions, reinforcement, such as rewards, incentives and punishment)	Environment/competing demands for HF management	“See, my dad had died from mesothelioma in 2011. He worked in a shipyard and I worked in the shipyard also about 12 years, and I used to work around all that stuff, the asbestos and stuff. I’m scared I might have some of that, not from my dad, but I worked in the same spots he did, and that kind of scared me because I know few people that I grew up with that worked with me, they’re dead now. And my dad died and all his friends died, and they all worked together.”—ptid129 “You know what, I—one thing that I—it really hurts me a lot, but it’s like I’m unable to work as before. It really affects me. That’s one main thing that I—I’m like, “Wow, that’s”—not being able to be the same person like before, like……able to work.”—ptid395 “Because I’m very involved with various community organizations. I provide consultancies to everything from Mission Language and Vocational School to the economic development and social services, mental health and everybody else that I’ve been involved with for several years now. So, I’ve had to curtail all of that. So, my answer in number one, extremely limited my enjoyment of life, yes, because I can no longer participate the way that I used to.” ptid400 “I’m a carpenter by trade. I can’t even do that a little bit now.”—ptid45
	Mental health: barriers	“It’s important for health care providers to understand that the physical toll is very great and lays heavy on a person, but as the emotional part of it is even harder to deal with.”—ptid107 “Emotionally, [heart disease] can—not all the time, but there are days when [heart disease] makes me depressed and scared, pensive, worried, anxious, afraid, angry.”—ptid107
*Reflective* (Beliefs about capabilities and consequences, roles, identity, intentions, goals, and optimism)	Mental health: resiliency	“Having just a doctor look you in your eye and telling you, “You have heart disease,” is very—excuse my word, disheartening. It’s hard. It’s very hard. I could see how some can throw in the white flag, but for me it motivated me to keep my head even higher.”—ptid375 “And now, you know, I’m—before, it was really hard for me to accept it that I’m having a heart failure and I was like, ‘Wow, I never thought this could happen to me.’ So, now those questions, it’s like now I look forward because I do try to answer, and I do—it doesn’t bother no more, but at first it would bother me because I didn’t wanna believe that I have a heart problem. I didn’t want to, you know, talk about it because it really bothered me. Like, I didn’t—now it doesn’t bother me at all. Now I won’t say I’m happy with this thing, but I’m learning to deal with this thing and then be more comfortable about it because before, it just put me more in the bag, more worries and things, now I’m learning day by day about my illness. I think I—I feel it’s something that I really like, especially with—talk—I talk with my doctors about it that I’m doing my best. I’m doing my best because it’s really had a major impact in my life and it’s—I have to continue. You know, I don’t want to—I wanna live. I wanna live. I know it’s not gonna be a normal life, a regular life, but I—I still…I’m trying as hard as I can to be…”—ptid395 “They don’t impact my life anymore. I’ve learned to let it go. Yes, I had a bout with depression when I first got diagnosed with this disease, but we don’t live like that anymore. I’ve learned to just let things just go into one ear and out the other, and not dwell on them, because if you sit there and you dwell on it it’s going to impact your heart way more than worrying about a little small, minute problem.”—ptid350 “I got my whole family—I haven’t seen nobody in my family in 3 years. My daughter don’t to speak to me. I’m all alone. So, this strength—the things I went through really taught me how to just be grateful and just pray on. But the Lord knows my heart, what I’m going through, and He’s been blessing me every day. So many good things happening to me now, and I’m just grateful that I turned my life around, and it just strengthened me, got me feeling better about myself.”—ptid129 “I had a change of lifestyle and I try to think differently and act differently too and try to push through whatever I think tries to limit me.”—ptid264

We calculated the FK reading level of the KCCQ-12 and it was higher than the recommended 8^th^-grade reading level for patient-reported outcome measures. Therefore, we adapted it to recommended reading levels and explored patient perceptions of the literacy-appropriate version (Table 4). While some patients found them to be “the same” (ptid29, ptid395), others preferred the simplified version. None of the interviewees expressed a preference for the original KCCQ-12 version on paper.

**Table 4:** Survey content

Q #	Original question	Readability (Flesch–Kincaid grade level)	Adapted question^a^	Readability (Flesch–Kincaid grade level)
1	Heart failure affects different people in different ways. Some feel shortness of breath while others feel fatigue. Please indicate how much you are limited by heart failure (shortness of breath or fatigue) in your ability to do the following activities over the past *2 weeks*.	9.1	How hard is it for you to do these activities because of your heart failure?	5.9
2	Over the *past 2 weeks*, how many times did you have *swelling* in your feet, ankles or legs when you woke up in the morning?	9.0	How many times do you wake up with swelling in your feet, ankles, or legs?	4.4
3	Over the *past 2 weeks*, on average, how many times has *fatigue* limited your ability to do what you wanted?	11.1	How many times do you feel too tired to do what you want?	2.1
4	Over the past *2 weeks*, on average, how many times has shortness of breath limited your ability to do what you wanted?	10.6	How many times do you feel too out of breath to do what you want?	2.8
5	Over the past *2 weeks*, on average, how many times have you been forced to sleep sitting up in a chair or with at least 3 pillows to prop you up because of shortness of breath?	12.8	How many times do you sleep sitting up or with pillows because you are out of breath?	5.6
6	Over the past *2 weeks*, how much has your heart failure limited your enjoyment of life?	6.8	How hard is it for you to enjoy your life because of heart failure?	4.1
7	If you had to spend the rest of your life with your heart failure the way it is right now, how would you feel about this?	7.2	Removed question based on recommendations for low-literacy audiences that “some individuals think in concrete/immediate rather than abstract/futuristic terms” *Clear and Simple*. Bethesda, MD: National Institutes of Health; 2016. Available at: https://www.nih.gov/institutes-nih/nih-office-director/officecommunications-public-liaison/clear-communication/clear-simple	N/A
8	How much does your *heart failure* affect your lifestyle? Please indicate how your heart failure may have limited your participation in the following activities *over the past 2 weeks.*	9.5	Combined with question 1	N/A

^a^The following recall period statement is included at the top page of the adapted survey: ‘Please share how heart failure has affected your life *over the past 2 weeks*. Mark the answer that best applies to you.’

## Discussion

This prospective qualitative thematic analysis of patients’ perceptions of the KCCQ-12 in an integrated, urban safety-net health system demonstrated that while items on the KCCQ-12 were relevant to their symptoms, they did not adequately capture all the factors that affect daily life with HF, such as diet, sexual activity, or key SDOH such as mental health and social relationships (Additional file 2). However, for patients to successfully manage their heart disease, all three components of the COM-B system (capability, opportunity, motivation) are important. Importantly, HF patients participating in these interviews did not draw categorical distinctions between symptoms, such as the extent of their shortness of breath, and their self-management, such as their dietary restrictions. Opportunity domains, such as one’s built environment and social factors like the quality of patient-provider communication, may influence a patient’s opportunity to engage in self-management behaviors. Mental health, resiliency, and social support are patient-important factors that were not captured on the KCCQ-12 but may influence a patient’s motivation to manage their heart disease. This is particularly relevant in this diverse, publicly insured population who face increased medical and social complexity in everyday life [12]. Inclusion of a broader range of factors into HF PROMs may increase their relevance for diverse populations.

In our exploratory analysis, patients preferred an adapted version of the KCCQ-12, which reflected a lower literacy level, consistent with prior studies comparing higher vs. lower literacy patient-facing materials [17]. Modifying the KCCQ-12 literacy level for diverse and low-income populations may improve its performance in predicting HF outcomes. Before the literacy-adapted version of the KCCQ-12 can be used, further testing in diverse populations with HF will need to be performed.

The KCCQ-12, a novel and well-validated health-related quality of life assessment tool for patients with HF, is comprised of distinct domains: physical limitations, symptoms (frequency, severity, and change over time), self-efficacy and knowledge, social interference, and quality of life [8, 18]. The subjective perception of dyspnea varies greatly and impacts the quality of life [19], and the KCCQ-12 captures this. The KCCQ-12 does not, however, capture patients’ SDOH, which has also been shown to impact risk [20]. It has been shown that correlating physical and psychological symptom profiles are predictive of survival [21]. The multiple factors that impact these, from mental health to housing to food insecurity, are acknowledged but seldom incorporated into patient assessments and shared-decision making [11]. The current study further supports the imperative to prioritize and directly assess the broader SDOHs that impact patient experience, disease management, and ultimately survival.

Social determinants of health deeply impact patient experience and outcomes and are disproportionately present in patients with HF as compared to the general population. For example, depression is more common in patients with HF and portends a worse prognosis [22, 23]. Similar trends are seen with other SDOHs such as socioeconomic position [24], food insecurity [25], and unemployment [26]. Non-White patients tend to experience these SDOHs more commonly than white patients, are underrepresented in clinical trials, and have poorer outcomes [2, 3, 10, 27]. This study of almost exclusively non-White patients brings to the surface these multiple factors that impact the burden of living with heart failure, from polypharmacy to dietary recommendations to social support to sex. Assessing and understanding how an individual patient navigates HF management with the competing demands of life, health, and work is paramount to providing thoughtful, personalized, and appropriate care.

Using the COM-B framework [16], we identified several key issues for the self-management of heart failure congruent with prior research [28, 29]. The KCCQ-12 most heavily captures capability, particularly physical issues, and patients’ feedback in this domain primarily focused on its psychological components. Most notably, patients in this study articulated the underrepresentation of the opportunity and motivation domains. They do not separate their HF symptoms from either external factors or conscious and unconscious processes that prompt and guide behavior. Patient perspectives on the overlap between the opportunity and motivation domains highlight the critical influence that SDOH has on HF self-management. Attributes in the opportunity and motivation domains weigh heavily on those facing medical and social complexity and have not been fully explored to date. This finding highlights a challenge in the use of PROMs across settings. Well-validated tools such as the PHQ-9 unfortunately do not fully capture those additional social determinants of health beyond mental health that impact their heart failure experience. Thus, inclusion of such an assessment in conjunction with the KCCQ-12 still leaves gaps in SDOH assessment. For medical product performance, PROMs need only include the target symptoms for the device or drug. However, for clinical care, patients experience their illness in the context of their social vulnerabilities. PROMs developed for regulatory purposes may be insufficient to inform clinical decision-making without consideration of SDOH.

Several limitations exist in the current study. We conducted interviews in a single safety-net health system hospital limiting external generalizability. However, this study touches on key demographic groups not typically represented in clinical studies. Only English-speaking patients were included, which unfortunately excluded non-English speaking patients in the SFHN, most commonly Spanish and Cantonese. This is an area of ongoing study. We employed the KCCQ-12 rather than the original 23-item KCCQ, which does have an item on depression and an item on sexual function. Based on our patients’ feedback, though, inclusion of these items would not have sufficiently addressed those SDOH deficiencies described above. Finally, the COVID-19 pandemic necessitated that the final 6 months of the study be conducted remotely via telephone or video conferencing. Given the socioeconomic makeup of our patient population, this likely impacted who was able to participate. Shelter-in-place requirements increased patient isolation and impacted patients’ feedback of the KCCQ-12. COVID-19 has exaggerated health disparities [30–32], and the impact of PROM and SDOH can be further explored.

## Conclusion

Patients from a diverse, low-income, majority non-white population in a safety net health care system experience unique challenges and circumstances that impact their ability to manage disease. It is important that future studies continue to enroll such patients, as SDOH disproportionately affects them negatively and impacts public health outcomes. In this study, patients were receptive to the KCCQ-12 as a tool but perceived that it did not adequately capture their illness experience because it did not assess key SDOH such as mental health and social relationships that deeply impact their ability to manage HF. Further study on the intersection of PROMs and SDOH reporting could investigate how to best use standardized patient reporting in evaluating and managing HF in diverse, underserved populations.

## Supplementary Information

**Additional file 1: Table 1**. Participant Perceptions of KCCQ-12.

**Additional file 2: Table 2**. Patient-Reported Impacts on Quality of Life.

## Data Availability

All data generated or analyzed during this study are included in this published article.

## Abbreviations

HF: Heart failure
PROMs: Patient-reported outcome measures
KCCQ: Kansas City Cardiomyopathy Questionnaire
SDOH: Social determinants of health
SFHN: The San Francisco Health Network
ZSFG: Zuckerberg San Francisco General Hospital
FK: Flesh Kincaid
COM-B: Capability, Opportunity, Motivation Model of Behavior
