# Supplementary material for: Do patient-reported outcome measures measure up? A qualitative study to examine perceptions and experiences with heart failure proms among diverse, low-income patients
Source: J Patient Rep Outcomes. 2022 Jan 15;6:6. doi: 10.1186/s41687-022-00410-9 (PMC8760874; doi:10.1186/s41687-022-00410-9)
Supplement: Supplementary file 1 — Additional file 1: Table 1. Participant Perceptions of KCCQ-12. [file 41687_2022_410_MOESM1_ESM.pdf]

UNIVERSITY OF CALIFORNIA, SAN FRANCISCO
Interview Guide: Aim 1 – Qualitative Interviews

Study Title:
Improving Heart Failure Patient-Reported Outcome Measures

Part I: For the first part of the interview, please answer the demographic and background questions at the end of your consent document.

Part II: I will ask you some additional background questions.

1. Do you use a computer? Tablet? Mobile phone?
    a. PROBE: GO ONLINE TO USE THE INTERNET? TEXT? SMARTPHONE, INCLUDING APPS?

CONSIDER SKIPPING QUESTIONS 2-5 IF NOT TESTING TABLET

2. What is your experience, if any, using a tablet?
    a. PROBE: Own one, used one, seen one used
3. What kinds of things do you do on the computer or tablet? What kinds of things do you do on your phone?
    a. (PROBE: INTERNET, SCHOOL/WORK, GAMES)
    b. If not, WHY DON'T YOU USE THE INTERNET? DO YOU TEXT?
4. (If applicable) Which device do you use to go online *most* often?
5. A lot of people say they need their son or daughter to get them on the Internet, or that they can only do a few things on their own. Do you ever have help when you want to use the internet?
    a. (PROBE: FAMILY MEMBERS HELPING, CONFIDENCE WITH TECHNOLOGY)
6. Can you tell me about a typical day in managing your health?
7. How does heart disease impact your everyday life?
8. What are the most important things you do to manage your health? What are the most important things you do to manage your heart disease?
9. Have you ever received a survey asking you about your health? If so, what did you think about it?
    a. PROBE: Did you complete the survey? Why or why not?
10. What do you think is important for health care providers to understand about how heart disease affects your life? Why is this important to you?
11. Do you think health care providers understand how heart disease affects your life?
    a. If yes, what do you think they understand? Why is this important to you?
    b. If no, what do you think they don't understand? Why is this important to you?

12. Have you ever had an experience where you felt like your health care providers did not understand your heart disease symptoms? What did they not understand? What do you wish they had done differently?
13. Have you ever had an experience where you felt like your health care providers did not understand your desired care for your heart disease? What did they not understand? What do you wish they had done differently?
14. Could you tell me how physical illness or injury has impacted your life in the last 30 days?
15. Could you tell me about how stress, depression, or other negative emotions have impacted your life in the last 30 days?
16. Would you say that poor physical or mental health has kept you from doing your usual activities, such as self-care, work, or recreation? Please describe.
17. In the last seven days, how many times did you miss taking your medications exactly as your doctor prescribed?
18. Some clinics are giving patients the option to complete surveys on the computer or mobile device. Is this something you would be interested in doing?
    a. PROBE: If yes, why are you interested in this? If not, why not?
    b. Would you rather complete a survey on paper or on a computer or mobile device?

Part III: We're now moving into the part of the interview during which we will talk about the survey.

INTRODUCTION

A patient-reported outcome measure is a survey that helps health care providers and researchers learn more about patients' health by asking patients directly, rather than using tests or exams. We are trying to learn how to design a survey that will make it easier for patients to share their thoughts about how heart disease affects their lives.

We are not testing or judging you on how well you can complete the survey or use a tablet, but trying to understand how easy or difficult using this survey is. There are no right or wrong answers. We are interested in how you use the survey to report how heart disease affects your life – which is why we are recording you and asking you to tell us what you're thinking as you go. I am trying to improve the survey, so you aren't hurting my feelings if you criticize it. My job is to find out what's wrong with it or where it can be improved. If you think the survey is confusing or difficult to use, that is extremely helpful for us to know so that we can make it easier for everyone to use. We appreciate your honest, positive and negative feedback.

Participants will be asked to complete 2 versions of the KCCQ survey:

1. *Tablet-based survey*
- ☐ Turn on tablet screen recorder

2. *Paper-based survey*

(RANDOM ORDER OF WHICH WILL BE COMPLETED FIRST)

PRACTICE EXERCISE

We're framing the interview today as a "think aloud" interview. This means that as you're doing the surveys, feel free to think out loud and let us know your thoughts as much as you feel comfortable doing. To get us in the mind frame of thinking out loud, I thought we could do a practice exercise:

"Try to visualize the clinic where you see your primary care doctor and think about the furniture, windows, and other things in the waiting room. As you count up the things in the room, tell me what you are seeing and thinking about."

Give positive feedback about things they described very clearly. Tell them to continue that level of detail if possible when using the surveys.

DESCRIPTION OF INTERVIEW PROCESS:

You will do two surveys, speaking aloud with any comments or questions as you answer the questions just like we did in the practice exercise. Then, we will go back to each survey and talk about the specific questions more.

Neutral prompts for the entire discussion

That's interesting, could you say a bit more about that?

What are you thinking now?

What makes you say that?

Could you tell me more about that?

Why do you think that?

What do you think about that?

Questions to ask after each KCCQ item

1) Did you understand the question?
2) Do you think this question is important in your everyday life?
3) Can you repeat this question back to me in your own words?
4) Do you think the words in the question need to be changed?
    a. If yes, how would you change it?
5) Do you think the responses should be changed?
    a. If yes, how would you change them?
6) Does this question relate to you and your experience with heart disease?

If applicable, questions to ask after each KCCQ section:

1) Did you understand the directions?

<u>AFTER EACH SURVEY (PAPER AND TABLET)</u>

Overall format-specific questions

How easy or hard did you think this survey was to complete?

What did you think about how the survey looks?

Is there anything you would change about the way the survey looks? (e.g. colors, visuals)

What do you think about the size of the letters?

Is there anything you would change about the way the survey works?

TABLET ONLY, IF APPLICABLE: How useful is the progress indicator to you?

TABLET ONLY, IF APPLICABLE: Do you think this progress indicator would be better? Why?

Would you have to ask someone to help you complete this survey?

Overall Perspectives

How familiar are you with questions/surveys like this about your heart disease? Had you seen these questions before?

Did the questions represent your overall experience dealing with heart disease?

Is there anything else that is important about your care that the survey doesn't ask you about?

Is there any question that should be removed from the survey?

Would you use this survey to report how heart disease affects your life?

> Probe: Why or why not? (when discussing the patient's overall quality of life in relation to the survey, return to relevant questions e.g. if they mentioned shortness of breath, go back to the associated questions).

<u>Overall Preferences</u>

- Thinking about the two versions of the survey you did, which one do you prefer?
