# Supplementary material for: Do patient-reported outcome measures measure up? A qualitative study to examine perceptions and experiences with heart failure proms among diverse, low-income patients
Source: J Patient Rep Outcomes. 2022 Jan 15;6:6. doi: 10.1186/s41687-022-00410-9 (PMC8760874; doi:10.1186/s41687-022-00410-9)
Supplement: Supplementary file 2 — Additional file 2: Table 2. Patient-Reported Impacts on Quality of Life. [file 41687_2022_410_MOESM2_ESM.docx]

**Table S1. Participant Perceptions of KCCQ-12**

| **Theme** | **Illustrative Quote** |
| --- | --- |
| **Ease of completing the KCCQ-12** | “Survey’s pretty easy to complete.” – ptid133  “[I] would say they were easy. I mean, very easy because these are – these questions, once again, they’re related with my heart condition, the symptoms that I feel. So, for me – for me it was easy because the questions and answers, it’s for symptoms that I experience with my illness. So, for me, that’s why - I consider easy it in a way because there are symptoms that I deal with - I deal with them day and – day by day.” – ptid395  “Because as I read the question, I turn around, start going with the answers right away. Because this is basically how you say – like an SAT test. […] Yes, you go right through it.” – ptid193 |
| **Attitudes about Completing the Survey in the Future** | “If they give you money, yes [I would complete the KCCQ in the future]. If without [Unintelligible] my voice, no, I don’t have time to spend it like this, I prefer to watch something funny.” – ptid327  “Well, I – I mean I don’t have a choice. I – I use whatever my doctor gives me. Whatever [Unintelligible] healthcare I am given, I mean that’s what I think. That’s what I have to [pick]. I mean sometimes I don’t know any where I’m just the patient.” – ptid264  “Yes – oh, one time. Or 10 times maybe, but not a thousand. Ten times maybe, not 100, but these surveys go around all the time.” – ptid29 |
| **Importance and relevance of the KCCQ-12 towards self-management behaviors** | “Yes, and I think it’s amazing that there is a survey and I think its follow-up is so important and it gives you a chance to look at your own measurements and reflect a little bit and then pass it on. You’re going to get all that information and you’ll see a pattern. It’s going to assist to see what’s what individually and as a group. I think it’s great that you would even do that.” – ptid51  “[…] I would use it because I – then I was - I would describe how – how - how I feel, how my symptoms are with my heart failure. So, for me, it would be a good way to describe my illness.” – ptid395 |

**Table S2. Reported Impacts on Quality of Life**

| **Theme** | **Illustrative Quote** |
| --- | --- |
| **Diet / Food** | “You could ask people how their heart failure has affected their choice of eating because it does affect your choice of eating. Like you’re sitting there you’ve got the coffee. I want a coffee but I can’t have a coffee. [Laughter] I mean there you go, you know? It affects you; you know? Diet is probably the most important thing. It’s probably more important than exercise.” – ptid350  “No. I mean there was nothing specific about dietary, and that’s always what they force you – not force but recommend for you to do.” – ptid396  “What I want to ask is – what I would ask is what are the things that you’ve had to curtail from your diet? What are the things you’ve had to curtail from your daily activities? And a lot of those, for the most part, are cultural-based answers. Some people have had to cut off chili, and not eating chili for the vast majority of cultures – of Latin culture is like a big – a big issue. It’s like eating Mediterranean food without sardines or having Italian food without pasta.” – ptid400  “Alcohol, drugs, and food. Those are the two main things that are the hardest that I have to do to better myself. I’m pretty sure I didn’t see them in there.” – ptid396 |
| **Prescription Medications** | “Yes, I do actually. I’m glad you asked that. Changes in medication, okay? That’s one of the things that – and it kinda, excuse me. It was one of the things that I kinda had questions about when I was doing my outpatient and some with inpatient, but outpatient, especially.” – ptid88 |
| **Sexual Activity** | “I guess it depends on how - what you’re going for, because it didn’t ask me about eating habits or drinking or sex or … I mean, it didn’t touch on a lot of subjects.” – ptid29  “I think so, [there should be a question about sex]. Because everybody who has heart trouble can’t have sex, male and female. They should ask about that.” – ptid190 |
